# Editorial: Current challenges and evidence-based medicine in psychiatric emergencies

**DOI:** 10.3389/fpsyt.2023.1145280

**Published:** 2023-03-01

**Authors:** Leonardo Baldaçara

**Affiliations:** ^1^Medicine, Federal University of Tocantins, Palmas, Tocantins, Brazil; ^2^Board of Directors, Associação Brasileira de Psiquiatria, Rio de Janeiro, Brazil

**Keywords:** psychiatric emergencies, mental disorder, suicide, psychomotor agitation, mental illness

Acute disruptions in cognition, behavior, mood, or social interactions that require immediate treatment and care to rescue the patient and/or others are psychiatric emergencies. Between 10 and 30% of all emergencies may be psychiatric emergencies (considering variations in each setting) (1). The percentage of the population (annual prevalence) that is afflicted by mental problems is anywhere from 8.6 to 57.3% when a public tragedy occurs (arises two-to three times more), with most cases being emergencies (2). However, in emergency settings, they are not always diagnosed correctly, and the consequences of mental illness are severe (3–5).

Suicidal behavior, severe depressive or manic episodes, self-mutilation, severely impaired judgment, severe self-neglect, substance intoxication or withdrawal symptoms, and aggressive behavior are among the most common psychiatric emergencies (3–6). Its consequences are devastating: physical (malnutrition, hydro-electrolyte disorders, trauma, and other physical complications), psychiatric (self-harm, suicide, substance abuse, aggression), and social consequences (social exposure, disappearance, and intensifying stigma) (3–5). It is unusual for students graduating from schools of health care to get instruction on psychiatric emergencies, and the majority of judgments are made based on non-technical and empirical data. This is due to the lack of research interest in the field, the effects of stigma, and the limited number of sectors with the necessary infrastructure to conduct studies and instruction (7, 8).

For instance, the total number of psychiatric consultations dropped during and after the lockdown, while the number of consultations for manic episodes and suicidality rose during this period. The study's authors argue that it's crucial to keep mental health care a priority, especially in the present, and prevent abandonment, negligence, and consequential new emergencies (Balestrieri et al.). In addition, the findings of another author revealed, despite the fact that they were not statistically significant, that psychiatrists who had been in practice for a longer period of time had a greater understanding of advanced directives. However, just a little more than a third of the participants were knowledgeable about this subject (Domingues et al.). It reinforces the need for teaching and training (7–9).

The United Nations Convention on the Rights of Persons with Impairments demands equal rights, non-discrimination, and social inclusion for those with disabilities and continues by outlining the manner in which the principles of human dignity, equality, non-discrimination, autonomy, and social inclusion apply to those who have disabilities. These demands equally apply to persons with mental illness. A rigid application of this statement in relation to involuntary hospital admissions may undermine the purpose of involuntary admission and treatment, which is to provide adequate mental health care to individuals whose mental disorders impair their ability to consent or refuse treatment. The purpose of involuntary admission and treatment is to provide adequate mental health care to individuals whose mental disorders impair their ability to consent or refuse treatment (Tamai).

A fascinating study looked at the top 100 cited articles on delirium to determine where the field stands and what global trends there are. The bibliometric research confirms the ever increasing interest in this topic area. With respect to delirium, the United States continues to lead the world. It is possible that researchers lean more heavily on cohort study findings. The findings also lend credence to further exploration (Fei et al.).

To finish, a research assessment assessed the main causes of preventable morbidity and mortality worldwide (4, 7, 10). Self-harm, also known as non-suicidal self-injury (NSSI), and suicide attempts are common reasons for adolescents to be sent to psychiatric hospitals for emergency care. It has been documented that adults who have a history of NSSI had altered baseline blood levels of β-endorphin (BE), but prior to this study, information on teenagers was missing. The authors conducted research on the psychoclinical profile as well as the serum BE level of 39 teenagers who had been hospitalized in the acute unit of a hospital in Spain because of NSSI. This discovery was not statistically significant, even though it showed that teenagers who appeared to be addicted to NSSI had greater levels of BE. It is necessary to do further research on the connection that exists between teenagers' blood BE levels and SIB (Wang et al.).

Then, in this Research Topic, we presented an add-on dedicated solely to EBM-based studies of psychiatric emergencies. Considering recent advances in our understanding of the origins, course, and remediation of mental diseases, it is crucial that our most extreme response strategies be modernized. All five of the featured masterpieces are of the highest caliber. Despite this, there remained a pressing need for fresh investigation that led to more efficient treatment for those with severe mental diseases.

## Author contributions

The author confirms being the sole contributor of this work and has approved it for publication.
